# Biomechanical evaluation of two modified intramedullary fixation system for treating unstable femoral neck fractures: A finite element analysis

**DOI:** 10.3389/fbioe.2023.1116976

**Published:** 2023-02-21

**Authors:** Qiang Huang, CongMing Zhang, HuanAn Bai, Qian Wang, Zhong Li, Yao Lu, Teng Ma

**Affiliations:** Department of Orthopedics, Hong Hui Hospital, Xi’an Jiaotong University, Xi’an, Shaanxi, China

**Keywords:** biomechanical, intramedullary, femoral neck fracture, axial, bending, torsion, finite element model

## Abstract

**Purpose:** The existing implants for fixation of femoral neck fractures have poor biomechanical stability, so the failure rate is high. We designed two modified intramedullary implants for treating unstable femoral neck fractures (UFNFs). We tried to improve the biomechanical stability of fixation by shortening the moment and reducing stress concentration. Each modified intramedullary implant was compared with cannulated screws (CSs) through finite element analysis (FEA).

**Methods:** Five different models were included: three cannulated screws (CSs, Model 1) in an inverted triangle configuration, the dynamic hip screw with an anti-rotation screw (DHS + AS, Model 2), the femoral neck system (FNS, Model 3), the modified intramedullary femoral neck system (IFNS, Model 4), and the modified intramedullary interlocking system (IIS, Model 5). Three-dimensional (3D) models of femur and implants were constructed by using 3D modelling software. Three load cases were simulated to assess the maximal displacement of models and fracture surface. The maximal stress at the bone and implants was also evaluated.

**Results:** FEA data showed that Model 5 had the best performance in terms of maximum displacement while Model 1 had the worst performance for this index under axial load of 2100 N. With respect to Maximum stress, Model 4 had the best performance while Model 2 had the worst performance under axial load. The general trends under bending and torsion load were consistent with that under axial load. Our data demonstrated that the two modified intramedullary implants exhibited the best biomechanical stability, followed by FNS and DHS + AS, and then three cannulated screws in axial, bending, and torsion load cases.

**Conclusion:** The two modified intramedullary designs showed the best biomechanical performance among the five implants included in this study. Therefore, this might provide some new options for trauma surgeons to deal with unstable femoral neck fractures.

## Introduction

Femoral neck fractures in young patients are usually caused by high-energy injuries, such as car accidents, falling height, etc. The treatment principle for such fractures is anatomical reduction and rigid internal fixation. Unstable femoral neck fractures, such as Pauwels type-III, bear larger shear stress, which is more prone to bone non-union and necrosis of femoral head than type I and type II (Knobe et al., 2018; Knobe et al., 2019). It is reported that the failure rate of fixation for UFNFs can be as high as 15–40% (Parker, 2000; Slobogean et al., 2015). The stability of implants is one of the important measures to ensure successful bone healing and avoid postoperative complications (Ragnarsson and Kärrholm, 1991; Ragnarsson and Kärrholm, 1992; Palm et al., 2009). As for biomechanical characteristics, important parameters such as stress and displacement are considered to affect the realization of good biomechanical stability. It is a challenge for trauma surgeons to improve biomechanical stability of existing implants. In recent years, seeking more effective implants to treat UFNFs has become a research hotspot in this field.

At present, the fixation methods of femoral neck fractures mainly focus on several extramedullary fixation devices, such as cannulated screws, the dynamic hip screw with an anti-rotation screw, and femoral neck system. The fixation method of multiple CSs is less traumatic and easy to insert (Blomfeldt et al., 2005; Lee et al., 2007). Yet, the use of CSs is prone to femoral neck shortening, screw withdrawal, cutting out, etc (Parker, 2009). Dynamic hip screw with an anti-rotation screw can play a better role in anti-rotation and anti-shearing than CSs, but it brings greater trauma (Schwartsmann et al., 2018). Other scholars inserted an medial plate to improve stability and shear resistance (Ye et al., 2017; Zhuang et al., 2018). However, the insertion of the medial auxiliary plate will inevitably lead to greater trauma and damage the blood supply of the femoral head. FNS has been used to fix femoral neck fractures in recent years, but its efficacy remains to be further observed (Stoffel et al., 2017). In short, the above implants are extramedullary fixation devices. They have a long moment, which is easy to cause stress concentration and eventually lead to fixation failure. As far as we know, although intramedullary implants are superior to extramedullary implants in biomechanics, there are few studies about intramedullary implants for treating UFNFs.

**FIGURE 1:**
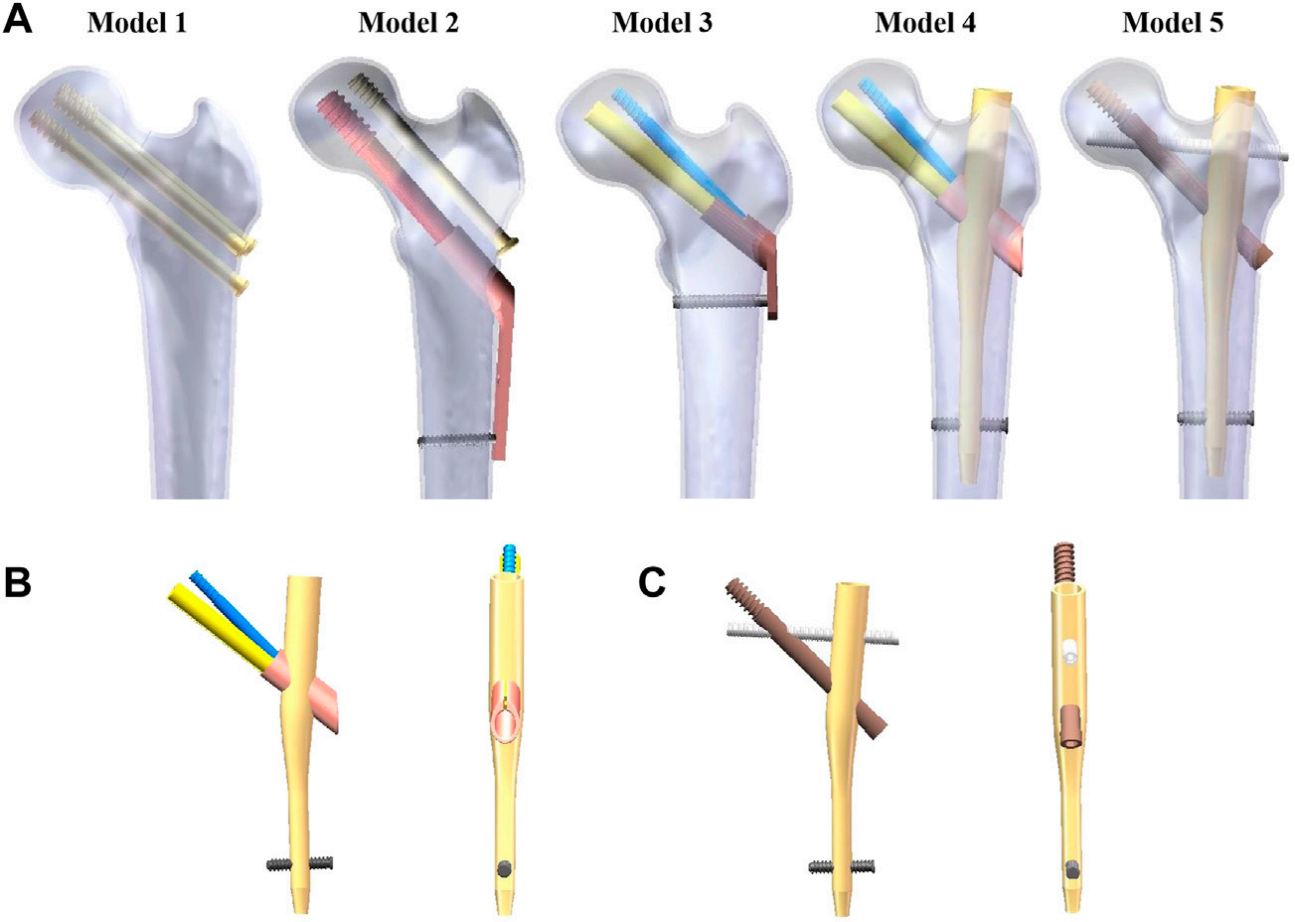
Five different implants after assembly of the finite element model. **(A)** Fixation of five different implants in the UFNF. **(B)** Schematic diagram of the modified intramedullary femoral neck system. **(C)** Schematic diagram of the modified intramedullary interlocking system. UFNF stands for unstable femoral neck fracture.

In view of this, the authors designed two modified types of intramedullary implants, specifically for the treatment of UFNFs. As shown in Figure 1, the proximal part of the two intramedullary implants are designed with two screws. Two neck screws pass through the fracture line at a specific angle. This design can meet the requirements of fracture anti-compression and anti-rotation. When the femoral head bears the stress in different directions, the stress can be transmitted to the main nail with a short moment and distributed along the femoral bone marrow cavity.

The biomechanical research of orthopedics adopts many methods, such as animal test (*in vivo*), physical modeling, cadaver model (*in vitro*), and computational simulation (*in silico*) through finite element analysis. Each research method has its own characteristics, including advantages and disadvantages (Hu and Wang, 2017). FEA is a numerical analysis method to simulate the real physical system by using mathematical approximation. This method can provide biomechanical evaluation and prognosis for various diseases and injury types, implant fixation and surgical techniques by tailoring finite element settings, such as material properties and boundary conditions (Leonardo-Diaz et al., 2020). It can not only make detailed quantitative estimation of displacement, but also quantitative estimation of load distribution in simulated surgical implants and surrounding bones. FEA has received extensive acceptance in the field of orthopedic research (Jitprapaikulsarn et al., 2021; Chantarapanich and Riansuwan, 2022). In this study, we designed two modified intramedullary implants for treating UFNFs. Each modified intramedullary implant was compared with cannulated screws through finite element analysis. The maximum stress and displacement on the bone and implants were evaluated. Three load cases were considered in the analyses which were axial, bending, and torsion loads. This study aimed to provide some new options for patients with UFNFs.

## Materials and methods

### Reconstruction of bone through 3D modelling and virtual surgery

The ethics committee of the Xi’an Hong Hui hospital approved this study (No.202202002). We recruited a 26-year-old healthy male volunteer with no history of hip joint or systemic diseases. The participant provided written informed consent to participate in this study. Computed tomography (CT) data images were collected to reconstruct 3D model of cancellous and cortical bones of femur *via* Mimics software (Materialise, Leuven, Belgium). CT examination uses X-ray to scan the body structure of the examiner, which will cause minimal radiation damage to healthy individuals, but usually will not cause serious damage. The CT (SOMATOM Definition AS1; Siemens, thickness, 0.6 mm; resolution, 512 × 512 pixels) scanning voltage and current were set in the range of 70–140 kV and 30–800 mA, respectively, until clear images were gotten. Then, CT images with the axial slice thickness of 1.5 mm were captured. We used Hounsfield Unit (HU) value (representing bone density threshold value) to distinguish cortical bones. The HU values for cancellous bones were defined as of less than 700, while that of cortical bones was more than 700 (Abdul Wahab et al., 2020). After that, a femoral neck fracture model with Pauwels angle of 70° was established. Computer-aided design (CAD) software (SolidWorks software, Dassault Systemes SolidWorks Corp., USA) was used to build five configurations of implants for UFNFs. The above fixation models were converted into the stereolithography (STL) format and exported to the 3-Matic software (Materialise, Leuven, Belgium), whereby the implants were inserted into the femur bone. Five different constructions were included: three CSs in an inverted triangle configuration (Model 1), DHS + AS (Model 2), FNS (Model 3), the modified IFNS (Model 4), and the modified IIS (Model 5). Figure 1A shows the five fixation models constructed in our study. Figures 1B, C show the two modified intramedullary devices. The dimensions of the implant designs were provided by the relevant manufacturer. For Model 4 and 5, the diameter and length of the main intramedullary nail is 10 mm × 170 mm. The diameter and length of two neck screws and one sleeve are 10 mm × 65 mm, 6.4 mm × 70 mm, and 14 mm × 35 mm for Model 4 while the diameter and length of two neck screws are 10 mm × 95 mm, 6.4 mm × 80 mm for Model 5, respectively. In Model 4, the included angle of the thick neck screw and the main nail is 130° while the included angle of the two neck screws is 7.5°, respectively. In Model 5, the included angle of the thick neck screw and the main nail is also 130° while the included angle of the two neck screws is 60°, respectively.

### Finite element setup

**FIGURE 2:**
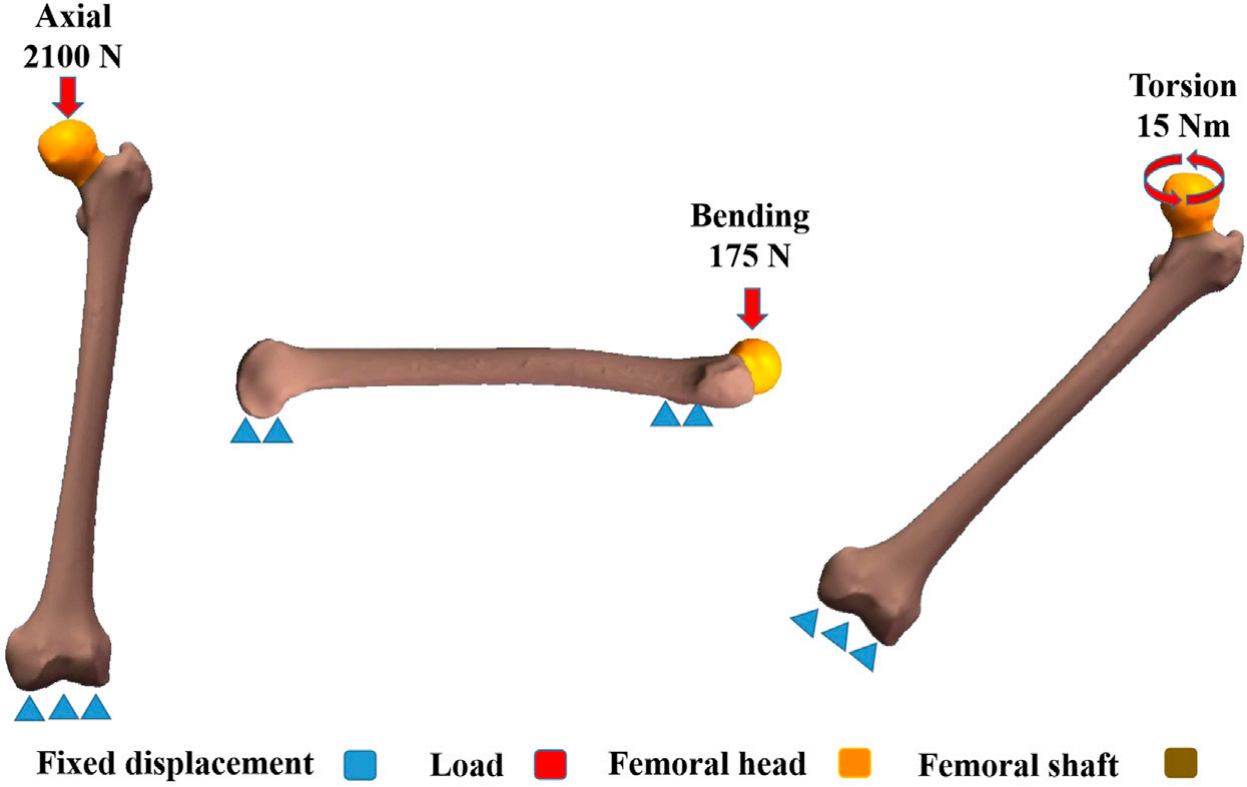
Boundary condition.

All three models had homogeneous and linear isotropic material features and were meshed with tetrahedral elements. In order to ensure the reliability of these models, a convergence study was conducted (Machado et al., 2014; McCartney et al., 2018). With maximum Degree of Freedom, field variables, such as strain energy and displacement, were within 5% for both types of elements and there was no maximum stress point. As shown in Table 1, with respect to material properties of the bones, the Young's modulus was defined as 16,800 MPa for cortical bones, 840 MPa for cancellous bones, and 110,000 MPa for implants while Poisson's ratio was 0.3 for cortical bones and Titanium alloy, and 0.2 for cancellous bones according to the literature (Li et al., 2018a). Table 2 showed the numbers of elements and nodes of five different fixation models. The implants were assigned with titanium material properties. All contact status was defined as frictional contacts, including the contact between fracture fragments and the contact between bone surface and implants. The friction coefficient of 0.4 was determined according to previous literature (Viceconti et al., 2000). As shown in Figure 2, three load conditions were simulated, including axial, bending, and torsion loads. In order to prevent rigid body movement during the analysis, the distal femur was fixed in all directions. In the axial load case, a 2,100 N load was acted axially on the femoral head representing the axial load compression (Zhang et al., 2011; Li et al., 2019). The load was exerted at the top of the femoral head. For the boundary of the torsion load case, a 15 Nm torsion load was acted on the surface of femoral head along the axis of the femoral neck and this represented the maximum load applied to the femoral head during normal human gait (Li et al., 2018b). As shown in Figure 2, in the bending load case, both femoral head and shaft of the femur were fixed in all degrees of freedom and a 175 N load was acted laterally on the femur from the front to simulate the four-point load bending (Li et al., 2018a). Marc Mentat (MSC Software, Santa Ana, CA) software was used for all finite element analysis and the analysis was solved by an implicit solver.

**TABLE 1:** Properties considered for the materials.

Material	Elastic modulus (MPa)	Poisson’s ratio
Cortical bone	16,800	0.3
Cancellous bone	840	0.2
Titanium alloy	110,000	0.3

**TABLE 2:** Number of nodes and elements for the five different models.

Model	Nodes	Elements
Model 1	1009970	662678
Model 2	1003617	679278
Model 3	1120474	782673
Model 4	1248368	845866
Model 5	1282546	861771

Model 1: three cannulated screws in an inverted triangle configuration. Model 2: the dynamic hip screw with an anti-rotation screw. Model 3: the femoral neck system. Model 4: the modified intramedullary femoral neck system. Model 5: the modified intramedullary interlocking system.

### Parameters for analysis

The maximal displacement of models and fracture surface was assessed under axial, bending and torsion loads. The maximal stress of implants and bone for five models were also evaluated. All values were compared with the value of Model 1. Model 1 (three CSs in an inverted triangle configuration) was considered to be the control group as this implant had the most extensive clinical application over the past years and was considered to have good biomechanical stability (Kauffman et al., 1999; Haidukewych et al., 2004). The corresponding variation rates were calculated by the following formula: VR =(V_1_ − Vn)/V_1_×100%, where VR = variation rate, Vn = value for Model 2, 3, 4, or 5, and V_1_ = value for Model 1.

## Results

### Maximal displacement of five models

**FIGURE 3:**
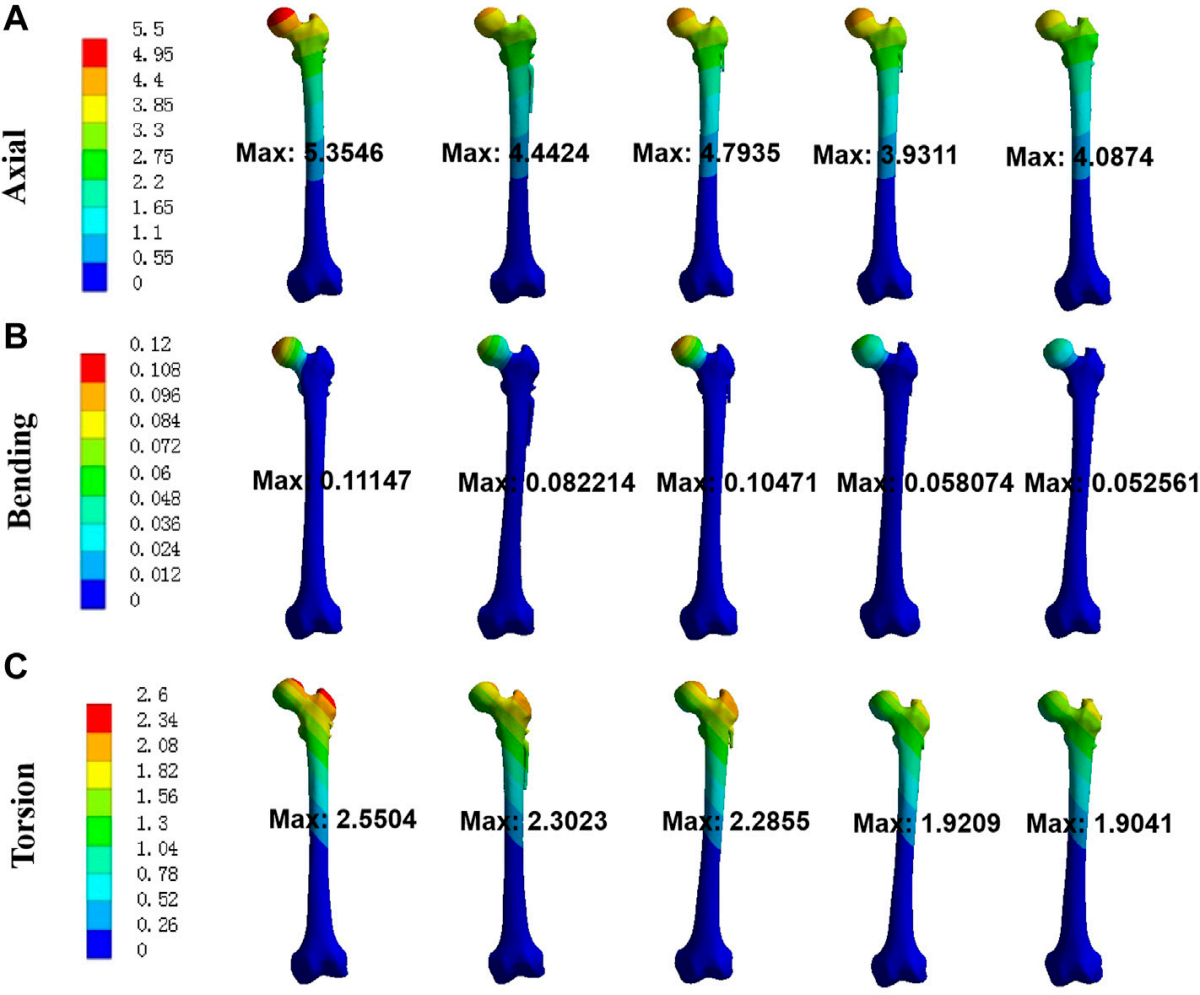
Maximal displacement of five models for three different loads. **(A)** axial; **(B)** bending; **(C)** torsion.

Maximal displacement of five models under axial, bending, and torsion load was shown in Figure 3. In order of the largest to smallest displacements under axial load of 2100 N, the five different fixation models were rated as follows: Model 1-3-2-5-4. For bending load of 175 N, it was rated as follows: Model 1-3-2-4-5. For torsion load of 15 Nm, it was rated as follows: Model 1-2-3-4-5. The maximum displacement of UFNFs fixed by the two modified intramedullary devices was smaller than that of CSs. The maximum displacement reduction of Model 4 and 5 relative to Model 1 reached 26.6% and 23.7% under axial load, 47.9% and 52.8% under bending load, 24.7% and 25.3% under torsion load, respectively.

### Maximal displacement of fracture surface for these models

**FIGURE 4:**
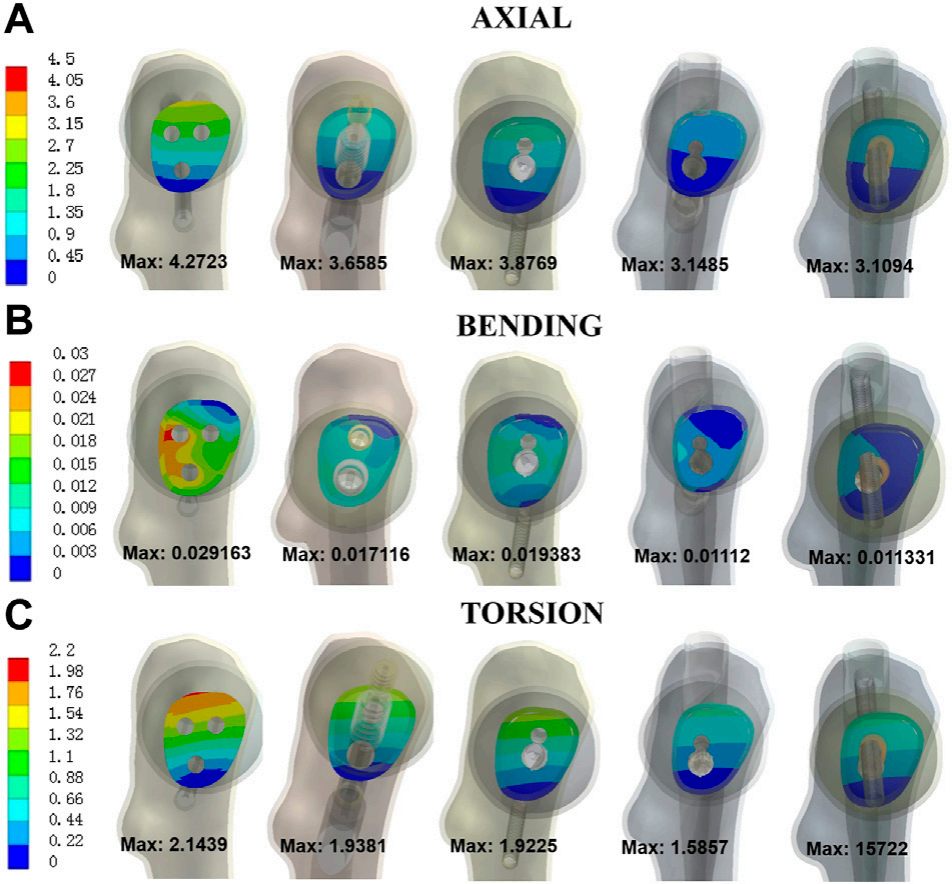
Maximal displacement of fracture surface for five models under three different loads. **(A)** axial; **(B)** bending; **(C)** torsion.

The trends of maximum displacement of fracture surface were consistent with that of maximum displacement of models (Figure 4). The maximum displacement of fracture surface from Model 1 to 5 was 4.27 mm, 3.66 mm, 3.88 mm, 3.15 mm, and 3.11 mm under axial load, respectively. Compared with Model 1, the maximum displacement reduction of fracture surface for Model 4 and 5 was 26.3% and 27.2% under axial load. Moreover, under bending load, the maximum displacement of fracture surface was 0.029 mm, 0.017 mm, 0.019 mm, 0.011 mm, and 0.011 mm for Model 1 to 5 while under torsion load it was 2.14 mm, 1.94 mm, 1.92 mm, 1.59 mm, and 1.57 mm, respectively. The maximum displacement reduction of fracture surface for Model 4 and 5 relative to Model 1 was 61.9% and 61.1% under bending load while 26.0% and 26.7% under torsion load. The above results showed that the modified intramedullary implant had better biomechanical stability than CSs for treating UFNFs.

### Stress at implants

**FIGURE 5:**
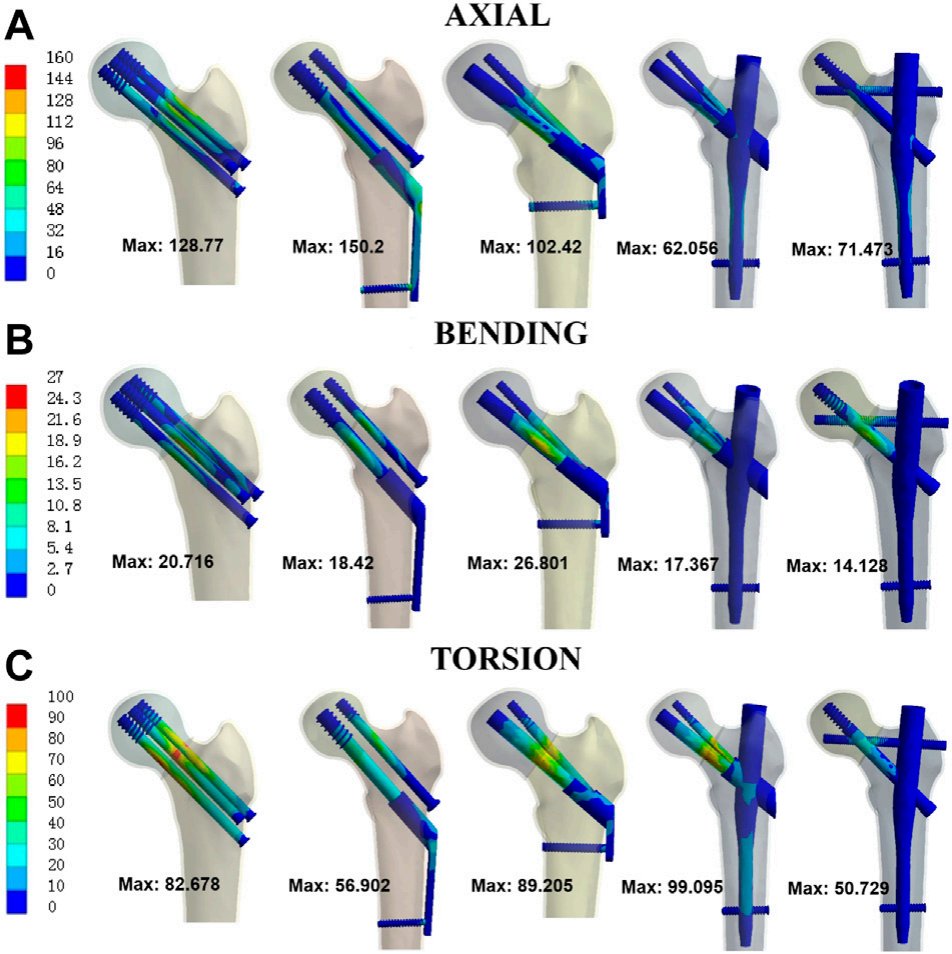
Von Mises stress at implants for three different loads. **(A)** axial; **(B)** bending; **(C)** torsion.

Stress at implants was shown in Figure 5 for five different models. In order of the highest to lowest maximal stress under axial load, the five different fixation models were rated as follows: Model 2-1-3-5-4. For bending load, it was rated as follows: Model 3-2-1-4-5. For torsion load, it was rated as follows: Model 4-3-1-2-5. Except Model 4 under torsion load, the maximum stress of each modified intramedullary fixation implant is less than that of CSs, respectively. The maximum stress reduction of Model 4 and 5 relative to Model 1 was 51.8% and 44.5% under axial load while 16.2% and 31.8% under bending load, respectively. Besides, compared with Model 1, the maximum stress reduction of Model 5 was 38.6% under torsion load.

### Stress at bone

**FIGURE 6:**
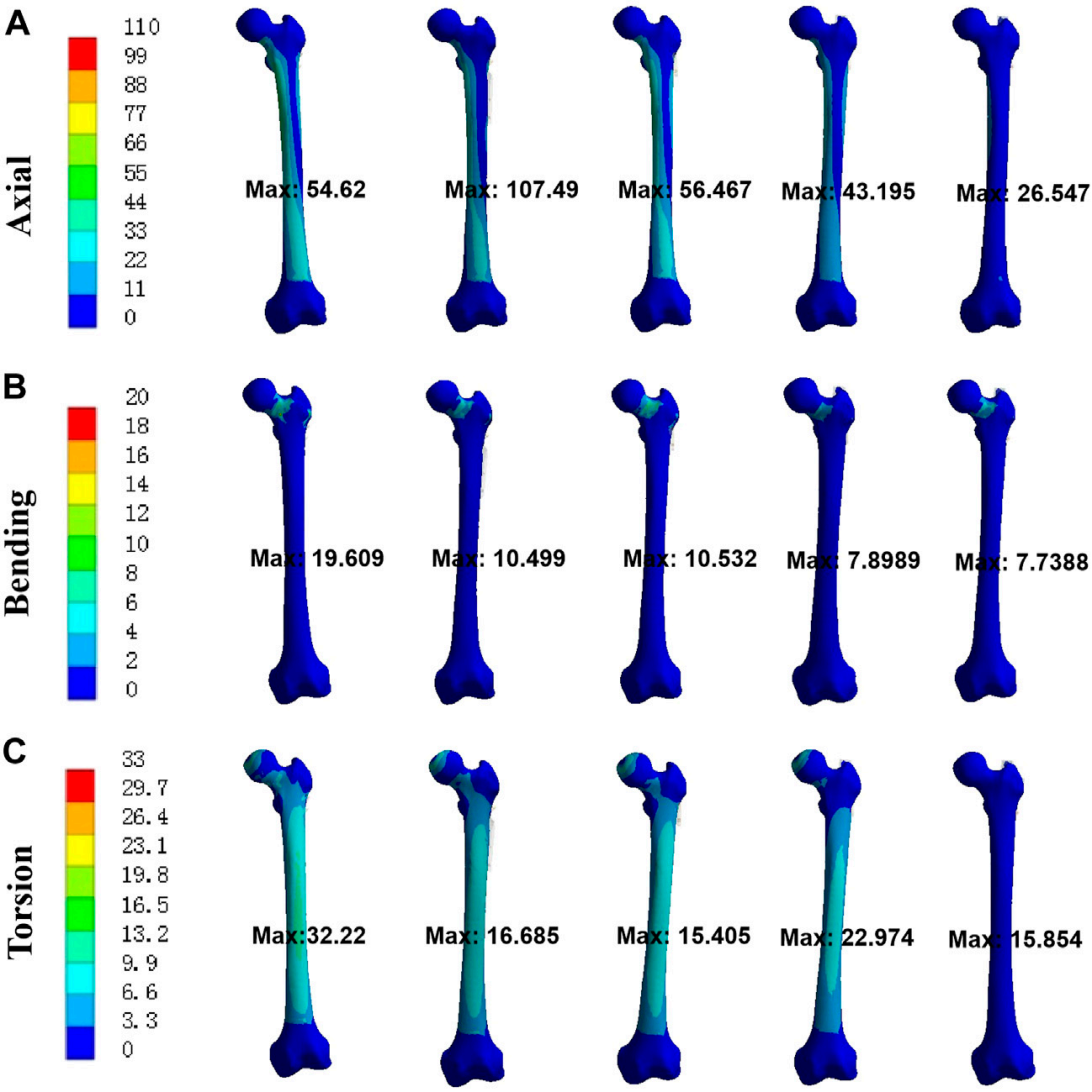
Von Mises stress at bones for three different loads. **(A)** axial; **(B)** bending; **(C)** torsion.

As shown in Figure 6, the trends of stress at bone were consistent with that of stress at implants for UFNFs. The maximum stress at bone from Model 1 to 5 was 54.620 MPa, 107.49 MPa, 54.467 MPa, 43.195 MPa, and 26.547 MPa under axial load (19.609 MPa, 10.499 MPa, 10.532 MPa, 7.8989 MPa, and 7.7388 MPa under bending load; 32.220 MPa, 16.685 MPa, 15.405 MPa, 22.974 MPa, and 15.854 MPa under torsion load). Compared with Model 1, the maximum stress reduction acted on bone for Model 4 and 5 was 20.9% and 51.4% under axial load (59.7% and 60.5% under bending load; 28.7% and 50.8% under torsion load). The above results indicated that each modified intramedullary implant had more uniform stress distribution than CSs for treating UFNFs, respectively.

## Discussion

Although the age range of hip replacement is wider than before, internal fixation is still the first choice for most young patients with femoral neck fractures. Weak blood supply and unstable fixation of the femoral head easily lead to non-union and necrosis of the femoral head after operation (Parker, 2000; Slobogean et al., 2015). Currently, surgeons focus on the extramedullary fixation for treating UFNFs, such as CSs, DHS, and FNS (Blomfeldt et al., 2005; Lee et al., 2007; Stoffel et al., 2017; Schwartsmann et al., 2018). Yet, there is no special intramedullary fixation device for the treatment of femoral neck fractures.

Several scholars have tried some modified techniques to fix UFNFs. Satish et al. used four CSs to fix unstable femoral neck fractures, in order to increase the whole stability (Satish et al., 2013). However, in clinical work it is difficult to arrange four CSs reasonably in cross section. Other scholars used three CSs and a medial auxiliary plate to fix such fractures (Ye et al., 2017; Zhuang et al., 2018). Although the stability of fixation has been significantly improved, this fixation method will increase trauma and even damage the blood supply of the femoral head in clinical work. FNS has been used clinically for several years, and some studies have shown that it has good biomechanical properties, and its performance is better than that of three CSs and DHS (Stoffel et al., 2017). Yet, FNS is an extramedullary fixation with a long level arm.

In view of this, some scholars explored whether intramedullary fixation of UFNFs can avoid the disadvantages of extramedullary fixation of such fractures. Guo et al. used intramedullary nails with cannulated screw fixation for the treatment of UFNFs (Guo et al., 2019). Their study showed that intramedullary fixation with cannulated screws had advantages for treating complicated femoral neck fractures in young and middle-aged patients. They used a combination of femoral reconstruction nails and cannulated screws in their study. However, it is difficult to insert the third or even fourth cannulated screw after the two cervical screws of the reconstruction nail have been inserted, especially when there is no robot assisted precise positioning or the patient’s femoral neck is thin. Röderer et al. investigated primary stability of the proximal femoral nailing anti-rotation (PFNA) for the indication of unstable medial femoral neck fractures (Röderer et al., 2011). They found PFNA achieved primary stability comparable to the dynamic hip screw blade. It also indicates that intramedullary fixation may be an option for the treatment of UFNFs. Yet, there is only one spiral blade at the femoral neck, and its anti-rotation ability is limited. Rupprecht et al. reported that compared with CSs and DHS, InterTan nail was more powerful for treating Pauwels type III femoral neck fractures (Rupprecht et al., 2011). Wang et al. conducted a biomechanical comparison of FNS, InterTan nail and three CSs for the treatment of Pauwels III femoral neck fractures (Wang et al., 2022). Based on their results, InterTan nail showed the highest axial stiffness and anteroposterior (AP) bending stiffness, followed by FNS, and then three CSs. However, the neck screws of InterTan nail are designed for parallel contact arrangement of two screws, which cannot achieve the most effective spatial distribution and fixation of the femoral neck section. In view of this, our team has tried to design two modified intramedullary fixation implants to better fix UFNFs.

Finite element analysis is a computer simulation method, which has been widely used to study implants for unstable femoral neck fractures (Peng et al., 2020; Zeng et al., 2020; Wang et al., 2022). It could evaluate the biomechanical properties of different implants by testing the stress and displacement data. We conducted finite element analysis of three CSs, DHS + AS, FNS, the modified IFNS, and the modified IIS for fixation of Pauwels type III femoral neck fractures. Our data demonstrated that the two modified intramedullary implants displayed the best biomechanical properties, followed by DHS + AS, FNS, and then three CSs under axial, bending, and torsion load. This is mainly because the two modified intramedullary implants have a short moment, which could provide enough mechanical strength and transfer the stress on the femoral head to the femoral shaft well. Besides, the intramedullary fixation is a central fixation, which makes the stress distribution of the internal fixation device uniform. There are two screws in the femoral head, which are distributed at a specific angle, so as to increase the anti-rotation effects. Both DHS and FNS are eccentric design, with long moment. In this way, the implants will bear relatively large compression and bending stress. Three CSs transfer torque through the interaction between the screws and the cancellous bones based on the “three-point support principle” (Wang et al., 2022). It should be emphasized that our modified IFNS is similar to classical FNS in design, but the difference is that we shorten the overall moment. This design is similar to the combination of an intramedullary nail and FNS. The finite element results also showed that the maximum displacement of the modified IFNS is less than that of FNS, and the same is true for the maximum stress of implants.

There are still some limitations to be further explored in this study. Firstly, in this research the cortical and cancellous bones were set isotropic, linear, and homogenous characteristics. In real-life, these bones own inhomogeneous characteristics (Couteau et al., 2001; Wong et al., 2021). To avoid excessive time consumption in the process of FEA modelling and considering that the current computer resources cannot be simulated using inhomogeneous models, thus isotropic and homogeneous characteristics were adopted. Secondly, three different loading conditions were simulated separately in our research. In reality, all three conditions might occur simultaneously while patients perform activities. It is another limitation of this research due to the limitation of computer resources at present and time consuming to simulate complex conditions. Further research needs to be carried out to simulate the abovementioned conditions which might provide some new insights to the understanding of biomechanical properties of patients treated by our modified implants. Thirdly, this research did not carry out model validation, which definitely is a common limitation of similar simulation studies. Yet, instead of accurate values of response, our team aimed to compare biomechanical properties and evaluate tendency on the basis of the same femur under the same loading and boundary conditions. As such, the lack of model validation might be justified to some extent. Many other scholars also applied the same method to simulate bones and implants with acceptable outcomes (Peng et al., 2020; Zeng et al., 2020; Wang et al., 2022). We will add the cadaver experiment in the further research, and finally apply the two modified implants to patients with unstable femoral neck fractures. Fourthly, when the two modified implants are used in clinical practice, the use of conventional size of proximal femoral rod may cause some new complications during operation, which needs further research.

## Conclusion

The two modified intramedullary designs possessed better biomechanical performance as compared to CSs in the axial, bending, and torsion load cases. Therefore, this might provide some new options for trauma surgeons to deal with unstable femoral neck fractures.

## Data Availability

The original contributions presented in the study are included in the article/supplementary material, further inquiries can be directed to the corresponding authors.
